# Influence of HER2 Changes on Survival Outcomes After Neoadjuvant Chemotherapy in Peruvian Patients With Triple-Negative Breast Cancer

**DOI:** 10.1155/tbj/3770655

**Published:** 2025-11-14

**Authors:** Zaida Morante, Yomali Ferreyra, Iris Otoya, Natalia Valdiviezo, Norma Huarcaya-Chombo, Gabriela Polo-Mendoza, Cindy Calle, Jessica Meza, Carlos Castañeda, Tatiana Vidaurre, Guillermo Valencia, Patricia Rioja, Hugo Fuentes, Silvia P. Neciosup, Henry L. Gomez

**Affiliations:** ^1^Faculty of Human Medicine, Cayetano Heredia Peruvian University, Lima, Peru; ^2^Department of Medical Oncology, National Institute of Neoplastic Diseases, Lima, Peru; ^3^Health Innovation Laboratory–Institute of Tropical Medicine “Alexander von Humboldt”, Cayetano Heredia Peruvian University, Lima, Peru; ^4^Professional School of Human Medicine, San Juan Bautista Private University, Lima, Peru; ^5^Faculty of Science and Engineering, Cayetano Heredia Peruvian University, Lima, Peru; ^6^Department of Bioengineering, University of Engineering and Technology, Lima, Peru; ^7^Oncosalud, Auna Ideas, Lima, Peru

**Keywords:** HER2-low, HER2-zero, neoadjuvant chemotherapy, prognosis, triple-negative breast cancer

## Abstract

**Background:**

Lack of human epidermal growth factor receptor 2 (HER2) expression limits targeted treatments for triple-negative breast cancer (TNBC). HER2 status changes after neoadjuvant chemotherapy (NAC) have been reported, but their impact on survival in Peruvian TNBC patients remains unexplored. Here, we aimed to assess HER2 status before and after NAC and its association with clinical characteristics, treatment response, and survival outcomes.

**Methods:**

Our analysis included clinicopathological data from 159 TNBC patients diagnosed between 2015 and 2019 at the Instituto Nacional de Enfermedades Neoplásicas (Lima, Peru) who received NAC. Logistic regression was used to assess the association between HER2 status at diagnosis and pathological complete response (pCR). Cohen's Kappa analysis evaluated the agreement between pre- and post-NAC HER2 status, while Kaplan–Meier analysis estimated the impact of HER2 changes on overall survival (OS) and disease-free survival (DFS)

**Results:**

Among TNBC patients, 40.3% were HER2-low at diagnosis and 14.9% achieved pCR. Pretherapeutic HER2 status was not associated with pCR (OR = 1.4, 95% CI = 0.55–3.61, and *p*=0.5). HER2 status remained unchanged in 62.8% of HER2-zero and 75.9% of HER2-low patients post-NAC, showing moderate concordance (Cohen's kappa = 0.3418, *p* < 0.001). No significant OS improvements were observed in patients with HER2 transitions: HER2-zero/HER2-low (HR = 0.52, 95% CI = 0.22–1.24, and *p*=0.14), HER2-low/HER2-zero (HR = 0.9, 95% CI = 0.34–2.40, and *p*=0.8), or HER2-low/HER2-low (HR = 0.71, 95% CI = 0.34–1.49, and *p*=0.4) compared with HER2-zero/HER2-zero. Similar findings were reported for DFS.

**Conclusion:**

These findings suggest that HER2 status conversion may not be prognostic for patients with TNBC treated with neoadjuvant therapy.

## 1. Introduction

Breast cancer (BC) is the malignant neoplasm with the highest incidence and mortality among women [1]. In this context, triple-negative breast cancer (TNBC) is characterized by the absence of estrogen receptor (ER), progesterone receptor (PR), and human epidermal growth factor receptor 2 (HER2). TNBC is well-known for lacking targeted therapy, which leads to an aggressive clinical profile ([2]; Han et al., 2023; [4]). It is associated with an increased risk of metastasis, residual disease, and poor prognosis among different populations ([2, 5]; Han et al., 2023), with an estimated 5-year overall survival (OS) rate of 56% in Peru [6].

TNBC primarily affects women under 60 years, particularly those with obesity or a BC family history [7–9]. Likewise, Black and Latino American women seem to have a higher risk than other populations [4, 7, 10]. One study identified a prevalence of TNBC of around 21.3% a single Peruvian public center [11].

Chemotherapy has been consolidated as the standard treatment for TNBC patients [2, 12]. Treatment strategies have shifted from upfront surgery followed by adjuvant systemic therapy to a preference for neoadjuvant chemotherapy (NAC), particularly in stages II-III [13]. NAC enables the evaluation of tumor response and the potential for personalizing postoperative treatment [14, 15]. A study focusing on TNBC patients in Peru indicated that younger and premenopausal patients, as well as those with advanced features (larger tumor size and nodal involvement), were more likely to receive NAC [6].

TNBC can be further categorized based on HER2 expression, such as HER2-zero or HER2-low. HER2-low tumors, defined as immunohistochemistry (IHC) 1+ or IHC 2+ with ERBB2 gene nonamplification, comprise approximately 32%–36% of TNBC cases [16, 17]. Although the prognostic value of HER2-low expression in TNBC, particularly in metastatic tumors, remains unclear, it has emerged as a potential target for new anti-HER2 drugs [16–18]. However, studies evaluating the impact of HER2 status changes on TNBC survival outcomes remain limited, particularly in Latin American populations. Thus, re-evaluating the HER2 status in patients with residual disease could be the key to identifying potential candidates for HER2-targeted therapies.

Our study aimed to evaluate the impact of HER2 receptor status conversion after neoadjuvant treatment on the survival outcomes of TNBC patients. Our objectives included analyzing the distribution of post-NAC clinical characteristics, the relationship between baseline HER2 status and treatment response, the conversion of HER2 status, and its influence on survival.

## 2. Methods and Materials

### 2.1. Design and Study Population

We retrospectively analyzed the clinical records of 982 patients with TNBC who were enrolled at the Instituto Nacional de Enfermedades Neoplásicas (INEN), Lima, Peru—the national reference center for oncology care in Peru—from January 1, 2015, to December 31, 2019.

Pretreatment samples were obtained through core needle biopsies performed during the diagnostic process. Post-NAC samples were derived from surgical specimens, which the pathology department at INEN routinely processed. All specimens were processed under standardized institutional protocols. IHC and fluorescence in situ hybridization (FISH) techniques were used to identify patients with TNBC. Antibodies Estrogen Anti-Receptor (Clone 1D5, Dako), Progesterone Anti-Receptor (Clone PGR 636, Dako), and Anti-HER2/neu (A0485, Dako) antibodies were used for IHC analysis [19, 20, 21].

### 2.2. Eligibility Criteria

We only included patients with a confirmed diagnosis of TNBC (stages I–III) over 18 years of age, who were treated with NAC (treatment time 3–10 months), and who underwent surgery.

We excluded TNBC patients treated or operated on at external institutions to avoid heterogeneity in treatment protocols, surgical techniques, and pathological assessments. Similarly, those with bilateral tumors were not considered to maintain cohort uniformity, as more complex clinical scenarios could introduce confounding factors.

**Figure 1:**
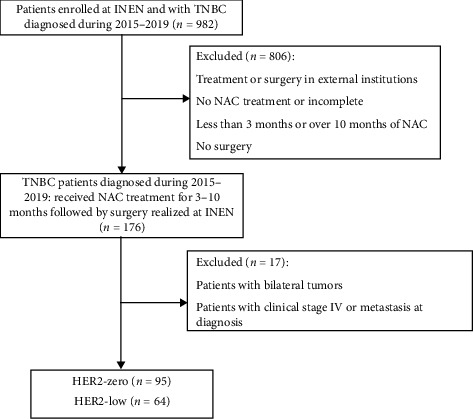
Flow diagram of study population and eligibility criteria.

Patients without HER2 status in the matched pre- and postoperative samples were excluded unless they had a documented HER2 status at diagnosis and a residual cancer burden (RCB) score. This process resulted in a population of 159 patients (Figure 1).

### 2.3. Definition of Variables

ER, PR, and HER2 status were determined according to the 2015 American Society of Clinical Oncology and College of American Pathologists (ASCO-CAP) guidelines. ER-negative and PR-negative were defined when ER < 10% and PR < 10%, which were used in routine clinical practice during the study period. FISH results corroborated the absence of HER2 protein overexpression. HER2 status was classified as HER2-low (IHC scores 1+ or 2+ with FISH negativity) or HER2-zero (IHC score 0) [22].

For clinical variables, age was considered as a continuous variable (years) and categorized into two approaches: three groups (≤ 40 years, 41–59 years, and ≥ 60 years) and four groups (≤ 40 years, 41–59 years, 60–69 years, and ≥ 70 years) to observe the distribution in the older population. Histology was categorized into ductal, lobular, mixed, or other. Clinical T stage (T1–T4) and clinical stages (II and III) were defined according to the American Joint Committee on Cancer (AJCC) Cancer Staging Manual, 6th edition. Histological grade was categorized as grade II (moderately differentiated) or grade III (poorly differentiated) according to the Elston–Ellis modification of the Bloom–Richardson system.

Ki67 index was expressed as a percentage and assessed at diagnosis and after NAC. The type of surgery was categorized as conservative or radical.

Pathological complete response (pCR) was defined as the absence of residual invasive tumor cells in the postoperative specimens from the breast and regional lymph nodes (ypT0/ypTis ypN0) [23]. RCB score was categorized as RCB-0 (pCR), RCB-I, RCB-II, and RCB-III.

Regarding treatments, NAC regimens were grouped based on drug composition: AC-T, AC-T + 5FU, AC/AC-T + Carboplatin + Taxane, and Carboplatin + Taxane. Less standard regimens, such as taxane monotherapy or TC, were classified as “Others.” Adjuvant chemotherapy regimens were categorized according to the use of capecitabine, which is considered the standard cytotoxic therapy for patients with residual disease following neoadjuvant treatment. Regimens without capecitabine, including continued taxane-based or platinum-containing therapies, were grouped as “Others.”

Disease-free survival (DFS) was calculated from the surgery date until the recurrence, occurrence of secondary malignancy, or death from any cause. OS was calculated from the date of surgery until death from any cause.

### 2.4. Data Collection

Medical records were reviewed to collect clinical data, including age, tumor size (in cm), clinical T staging, histological tissue types, clinical stages, histological grade, and IHC results (ER, PR, HER2, and Ki-67) before and after NAC.

We collected clinical data on patients, including their age, clinical T, expression of ER, PR, HER2, and Ki-67 index before and after NAC, RCB, surgical information, radiotherapy, post-operative therapy, relapse time, OS, and DFS.

For data extraction, a structured data collection sheet was created in Microsoft Excel, utilizing built-in data validation features to adhere to predefined variable categorizations and minimize input errors in the dataset. Any discrepancies were resolved through discussion or consultation with a senior investigator.

### 2.5. Statistical Analysis

For descriptive analysis, we used statistical tests, such as Kruskal–Wallis rank sum, Pearson's Chi-squared, and Fisher's exact, to report clinical characteristics (categorical variables) of patients and assess their association with the HER2 status at diagnosis. Logistic regression analysis was used to evaluate the association between the initial HER2 status and pCR.

Cohen's Kappa analysis was conducted to estimate the concordance of HER2 status at diagnosis and after NAC. This test was selected because it measures agreement beyond chance between two categorical assessments and provides a coefficient value (from 0 to 1) to quantify it. To interpret the results from this test, the level of concordance of HER2 transition was classified as poor, ≤ 0.2; mild, 0.2–0.4; moderate, 0.4–0.6; substantial, 0.6–0.8; and almost perfect, > 0.8 [24]. A univariable Cox proportional hazard model was used to estimate the prognostic implications of the HER2 status and other clinical variables. Kaplan–Meier and log-rank tests were conducted to assess DFS and OS and to compare the subgroups of HER2 changes. Statistical analyses were performed using the R software, Version 4.3.1. Statistical significance was set at *p*  <  0.05.

### 2.6. Ethical Considerations

The Ethics Review Board of INEN approved the study (INEN 24-23) and complied with all relevant ethical guidelines. Informed consent was not required, as the retrospective approach involved no risk to the subjects, and the database did not contain any information related to patient identity.

## 3. Results

### 3.1. Clinical Characteristics Among TNBC Patients

A total of 159 patients with TNBC who received NAC as their initial treatment were included. Among these patients, 59.7% (*n* = 95) were classified as HER2-zero at diagnosis, whereas 24.5% (*n* = 39) and 15.7% (*n* = 25) were classified as HER2-low, with HER2 IHC scores of 1+ and 2+, respectively. Median age of the overall population was 48.0 (interquartile range: 41.5, 55.5) years, with the most common age group being 41–59 years (58.5%, Table 1). An even distribution was also found among HER2 status: 23.2% of the patients were HER2-zero and 18.8% were HER2-low. A similar distribution was found for other age groups.

Most clinical characteristics showed no significant differences between patient groups with varying HER2 statuses. Histology revealed a predominance of the ductal type in all groups (91.6% in HER2-0 and 90.6% in HER2-Low, Table 1) compared with other types, such as lobular and mixed. Median tumor size remained consistent between both groups (*p*=0.3). Regarding clinical tumor staging, there was a higher proportion of T4 tumors in the HER2-low group (46.3% compared with 34.1%, Table 1). However, the difference was not statistically significant among HER2 statuses (*p*=0.4, Table 1).

Regarding the clinical stage, patients were predominantly stage III (54.7%), with no significant differences between groups (*p*=0.2). Histological grade level III was predominant among the HER2 statuses (87.0% for HER2-zero and 86.7% for HER2-Low, Table 1) with no significant difference (*p* > 0.9).

No significant differences were observed between Ki67% at diagnosis and HER2 scores (*p*=0.5, Table 1). Most patients received an AC-T–based regimen (71.1%), with similar proportions observed in both HER2-zero (71.6%) and HER2-low (70.3%) groups. Overall distribution differed significantly between HER2 groups (*p*=0.008, Table 1).

Regarding the type of surgery, radical surgery was the most frequent (74.2%), but there were no significant differences (*p*=0.3, Table 1). RCB scores varied between the HER2 score groups. HER2-zero patients mostly had RCB score II (42.9%), and RCB score III was predominantly present in HER2-low TNBC patients (50.9%). Approximately 85.1% of the patients did not achieve pCR (Table 1).

Capecitabine was the most frequently administered regimen (65.0%), and radiotherapy was administered to 83.0% of the patients. It was observed that 65.4% of TNBC patients remained alive and 69.2% did not present recurrence (Table 1).

### 3.2. Association Between HER2 Status and pCR

In our study, only 11.9% (*n* = 19) reached pCR (Table 1). Logistic regression analysis revealed that TNBC patients with a larger tumor size had a lower probability of achieving pCR (OR = 0.8, 95% CI = 0.61–0.94, and *p*=0.023, Table 2). Similarly, clinical stage level III vs. II was significantly associated with lower pCR events (OR = 0.3, 95% CI = 0.09–0.73, and *p*=0.013, Table 2). Ki-67 percentages (OR = 1.0, 95% CI = 0.98–1.02, and *p*=0.9, Table 2) and HER2 status at diagnosis (HER2-zero vs. HER2-low, OR = 1.4, 95% CI = 0.55–3.61, and *p*=0.5, Table 2) were not associated with pCR.

### 3.3. Evolution of HER2 Status After NAC

**Figure 2:**
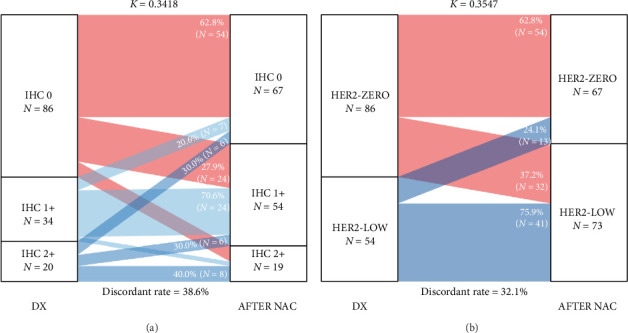
Changes in HER2 status before and after neoadjuvant chemotherapy. (a) IHC score-based transitions (0, 1+, 2+). (b) HER2-zero versus HER2-low classification transitions.

After NAC, 140 patients had residual disease classified as TNBC. No patient converted to ER- or PR-positive. Posttreatment assessment showed that 92.1% (*n* = 129) and 95.7% (*n* = 134) were ER- and PR-negative, respectively, while 4.3% (*n* = 6) and 3.6% (*n* = 5) exhibited low ER and PR expressions (Supporting Tables S1 and S2). Among those initially classified as HER2-zero (*n* = 86), 62.8% (*n* = 54) remained HER2-zero, whereas 37.2% (*n* = 32) were reclassified as HER2-low, specifically 27.9% (*n* = 24) changed to IHC 1+ and 9.3% (*n* = 8) to IHC 2 (Figure 2(a) and Supporting Table S3). Conversely, among those initially classified as HER2-low (*n* = 54), 75.9% (*n* = 41) maintained this status and 24.1% (*n* = 13) shifted to HER2-zero (Figure 2(b)).

Cohen's kappa values and discordant rates were 0.3418% and 38.6%, respectively, for the IHC scoring system (Figure 2(a)) and 0.3547% and 32.1% for the HER2 classification (Figure 2(b)). A statistically significant association was found between pre- and post-therapeutic HER2 status (*p* < 0.001, Supporting Tables S3 and S4). No clinical or treatment-related characteristics were found to be associated with HER2 conversion after NAC (Supporting Table S5)

### 3.4. HER2 Changes and Clinical Outcomes

**Figure 3:**
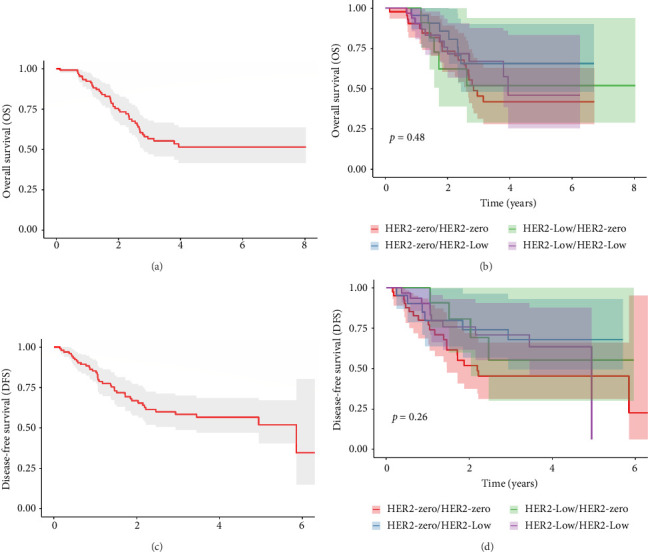
HER2 evolution and survival outcomes. (a) OS of all TNBC patients. (b) DFS of all TNBC patients. (c) OS by HER2 status change. (d) DFS by HER2 status change.

Median OS could not be reached; meanwhile, the median DFS was 5.85 years (Figures 3(a) and 3(c)). It was observed that TNBC patients who migrated from HER2-zero to HER2-low status, compared with patients with HER2-zero status who did not present changes, did not show a significant improvement in OS (HR = 0.52, 95% CI = 0.22–1.24, and *p*=0.14, Table 3) or DFS (HR = 0.48, 95% CI = 0.19–1.20, and *p*=0.12, Table 3). For patients whose HER2 status decreased from low to zero, the analysis showed no significant impact on survival outcomes (vs. HER2-zero/HER2-zero, OS: HR = 0.9, 95% CI = 0.34–2.40, and *p*=0.8; DFS: HR = 0.54, 95% CI = 0.18–1.58, and *p*=0.3, Table 3). Additionally, patients maintaining a low HER2 status throughout the study did not reach significantly better survival outcomes (vs. HER2-zero/HER2-zero, OS: HR = 0.71, 95% CI = 0.34–1.49, and *p*=0.4; DFS: HR = 0.58, 95% CI = 0.27–1.25, and *p*=0.2, Table 3). Other clinical characteristics were not significantly associated with OS and DFS (Supporting Table S6).

## 4. Discussion

Our study evaluated the impact of HER2 status conversion after NAC on DFS and OS in TNBC patients and found no significant effect on survival outcomes.

HER2 receptor assessment is standard practice in BC to guide treatment decisions [22]. TNBC, known for its molecular heterogeneity, lacks approved targeted therapies [2, 12, 25, 26]. In this context, HER2-low has become a relevant topic due to its potential therapeutic implications.

HER2 conversion after NAC is likely driven by biological mechanisms, particularly intratumor heterogeneity and clonal selection under chemotherapy, where treatment pressure eradicates certain HER2-positive clones while allowing HER2-negative or low subpopulations to expand [27]. Our results showed fair concordance in HER2 status after NAC. In comparison, a Korean TNBC cohort reported slightly higher agreement, with a moderate kappa value of 0.40 and a discordant rate of 25.6% [28]. Although HER2 status in TNBC is not entirely stable, it appears comparatively more consistent than in other subtypes. In the same study, HR-positive tumors exhibited a higher discordant rate (33.8%) and a lower kappa value (*k* = 0.31). This relative stability in TNBC was also supported by Xian et al. [29], who found that HER2 status was more likely to remain stable after NAC in TNBC compared with HR + patients (91% vs. 58%, *p* < 0.01).

However, evidence suggests these transitions may become more noticeable during disease progression, particularly in studies that include all BC subtypes. For example, Anderson et al. [30] observed that HER2-low to HER2-zero transitions were more frequent (43.2%, *p*=0.03) during tumor evolution, suggesting a potential role for tumor evolution under selective pressure.

Regarding treatment response, we found no significant association of HER2 status at diagnosis with pCR. A Brazilian study demonstrated that no differences in pCR rates were observed between TNBC, with 51% for HER2-low tumors and 47% for HER2-negative (*p*=0.64) [31]. Nonetheless, another study showed that HER2-low status was associated with a slightly reduced likelihood of pCR (OR = 0.89; 95% CI = 0.86–0.92; and *p* < 0.001) [32].

Survival outcomes in TNBC vary across populations. In India and China, 8-year and 7-year OS rates were 75% and 71.64%, respectively [33, 34]. In contrast, Peruvian TNBC patients exhibited lower survival rates, with 56% at 5 years and 47% at 10 years [6], which may reflect the influence of genetic ancestry and disparities in access to timely diagnosis or treatment.

Several studies suggest a potential survival benefit associated with HER2-low status at baseline in TNBC. A meta-analysis reported better rates of OS for HER2-low vs. HER2-zero TNBC (HR = 0.85 and 95% CI = 0.71–0.98) [35]. Similarly, a Korean study linked HER2-low with better breast cancer-specific survival (BCSS) (HR = 0.68; 95% CI = 0.49–0.93; and *p*=0.019) and improved 5-year DFS rates (76.4% vs. 65.5%, *p*=0.026) [36]. Also, stages II–IV TNBC from the United States showed HER2-low had a slightly higher 5-year OS compared with HER2-zero [32]. Similar trends have been observed in Europe. A German study reported significantly higher 3-year OS in HER2-low vs. HER2-zero (90.2% vs. 84.3%; *p*=0.016) and improved DFS (84.5% vs. 74.4%; *p*=0.0076) [16]. Furthermore, a French cohort noted better OS in HER2 IHC 2+ compared with IHC 1+ (*p*=0.042) alongside better DFS (*p*=0.037) [37].

However, some populations showed no survival differences. For Austrian and Portuguese populations, no significant differences in OS between HER2-low and HER2-zero (HR = 0.95; 95% CI = 0.79–1.13; and *p*=0.545) were found [17], while a Chinese study reported similar 5-year OS (90.6% vs. 89.8%; *p*=0.88) and DFS (81.9% vs. 80.1%; *p*=0.819) between these groups [38]. These findings suggest that the association of HER2-low with better survival outcomes may vary across populations.

Still, few studies have been published on how HER2 status changes impact survival outcomes among TNBC. Kang et al. [28] discovered that those BC patients who experienced HER2-low to HER2-zero transitions correlated with better OS (*p*=0.0009) and DFS (*p*=0.00028). However, subgroup analysis by HR status had no significant differences among HR-negative subsets for those with that transition in OS (*p*=0.29) and DFS (*p*=0.12). Our findings support this observation. Thus, HER2 status changes after NAC could clarify how tumor adaptation under selective pressure influences survival outcomes differently, potentially varying by subtype or across different populations.

In this sense, post-NAC rebiopsy in TNBC is not only helpful in evaluating residual disease but also for reassessing HER2 status, which may shift toward a HER2-low phenotype after treatment. HER2-zero to HER2-low transition could have potential clinical relevance in the context of emerging targeted therapies. In the Phase III DESTINY-Breast04 trial, trastuzumab deruxtecan (T-DXd) reduced the risk of death by 52% compared with standard chemotherapy among the TNBC HER2-low subgroup (HR = 0.48; 95% CI = 0.24–0.95; and *p*=0.0303) [39]. Despite the current lack of approval in early-stage disease, the observed benefit of T-DXd underscores the potential value of post-NAC HER2 reclassification in guiding treatment decisions for patients with residual TNBC.

## 5. Limitations

Our study has some limitations. First, the relatively small sample size may have been largely due to the exclusion of patients with unavailable matched pre- or postoperative HER2 status. Although this approach was necessary to compare HER2 transitions, it may not fully represent the general TNBC population of our cohort and may limit the statistical power to detect significant associations.

Another inherent limitation is that pre- and post-NAC receptor status assessments were performed on different specimen types. Although this reflects standard practice, it may introduce variability in IHC results due to tissue handling and heterogeneity. Nonetheless, this approach is widely accepted and endorsed by clinical guidelines and other studies.

Additionally, the retrospective nature of the study introduces potential biases, as data collection relied on medical records. We also did not include tumor-infiltrating lymphocytes (TILs) as a variable because the institution did not routinely report this parameter during the study period. Including TILs and other inflammatory biomarkers could provide insight into the evaluation of immune responses and their relationship with HER2 transitions in treatment response or survival outcomes in future studies.

However, it is essential to note that our research is one of the few to investigate changes in HER2 status after NAC, specifically in Latin American TNBC patients, who are often underrepresented in clinical research. Furthermore, using a standardized HER2 assessment based on ASCO-CAP guidelines ensures consistency and reliability in our findings. Despite these limitations, our study provides valuable insights into changes in HER2-low status, particularly among Latin American patients with TNBC.

## 6. Conclusion

Our study showed that conversion HER2 status after NAC did not impact survival outcomes nor did HER2 status at diagnosis relate to response to treatment. Further research and clinical trials are crucial for transforming the paradigm of TNBC treatment and exploring the potential role of biomarkers in enhancing the prognosis and quality of life for patients affected by this aggressive subtype.

## Figures and Tables

**Table 1:** Clinical characteristics by HER2 status.

Characteristic	Overall	HER2 0	HER2 low	*p* value^2^
	*N* = 159^1^	*N* = 95^1^	*N* = 64^1^	
Age (years)	48.0 (41.0, 56.0)	48.0 (41.0, 55.0)	48.5 (42.5, 57.0)	0.4
Age (years)				0.8
≤ 40	34 (21.4%)	22 (23.2%)	12 (18.8%)	
41–59	93 (58.5%)	55 (57.9%)	38 (59.4%)	
≥ 60	32 (20.1%)	18 (18.9%)	14 (21.9%)	
Age (years)				0.9
≤ 40	34 (21.4%)	22 (23.2%)	12 (18.8%)	
41–59	93 (58.5%)	55 (57.9%)	38 (59.4%)	
60–69	24 (15.1%)	14 (14.7%)	10 (15.6%)	
≥ 70	8 (5.0%)	4 (4.2%)	4 (6.3%)	
Histology				0.9
Ductal	145 (91.2%)	87 (91.6%)	58 (90.6%)	
Lobular	3 (1.9%)	2 (2.1%)	1 (1.6%)	
Mixed	5 (3.1%)	2 (2.1%)	3 (4.7%)	
Other	6 (3.8%)	4 (4.2%)	2 (3.1%)	
Tumor size (cm)	6.0 (4.0, 8.0)	6.0 (4.0, 8.0)	6.0 (4.5, 8.0)	0.3
Not reported	2	1	1	
Clinical T				0.4
T1	3 (2.2%)	3 (3.5%)	0 (0.0%)	
T2	38 (27.3%)	25 (29.4%)	13 (24.1%)	
T3	44 (31.7%)	28 (32.9%)	16 (29.6%)	
T4	54 (38.8%)	29 (34.1%)	25 (46.3%)	
Not reported	20	10	10	
Clinical N				0.7
N0	33 (28.9%)	22 (29.7%)	11 (27.5%)	
N1	55 (48.2%)	37 (50.0%)	18 (45.0%)	
N2/N3	26 (22.8%)	15 (20.3%)	11 (27.5%)	
Not reported	45	21	24	
Clinical stage				0.2
II	72 (45.3%)	47 (49.5%)	25 (39.1%)	
III	87 (54.7%)	48 (50.5%)	39 (60.9%)	
Histological grade				> 0.9
II	20 (13.2%)	12 (13.0%)	8 (13.3%)	
III	132 (86.8%)	80 (87.0%)	52 (86.7%)	
Not reported	7	3	4	
Ki67% (diagnosis)	60.0 (40.0, 70.0)	60.0 (40.0, 80.0)	60.0 (40.0, 70.0)	0.5
Not reported	10	2	8	
Neoadjuvant chemotherapy regimen				**0.008**
AC-T	113 (71.1%)	68 (71.6%)	45 (70.3%)	
AC-T + 5FU	4 (2.5%)	0 (0.0%)	4 (6.3%)	
AC/AC-T + carboplatin + taxane	25 (15.7%)	14 (14.7%)	11 (17.2%)	
Carboplatin + taxane	9 (5.7%)	9 (9.5%)	0 (0.0%)	
Others	8 (5.0%)	4 (4.2%)	4 (6.3%)	
Surgery type				0.3
Conservative	40 (25.8%)	27 (29.0%)	13 (21.0%)	
Radical	115 (74.2%)	66 (71.0%)	49 (79.0%)	
Not reported	4	2	2	
HER2 status (post-NAC)				**< 0.001**
HER2-zero	67 (47.9%)	54 (62.8%)	13 (24.1%)	
HER2-low	73 (52.1%)	32 (37.2%)	41 (75.9%)	
Not applied	19	9	10	
Ki67% (post-NAC)	60.0 (40.0, 70.0)	60.0 (40.0, 75.0)	60.0 (40.0, 70.0)	> 0.9
Not reported	34	15	19	
Residual cancer burden (RCB) score				0.053
0	19 (11.9%)	9 (9.5%)	10 (15.6%)	
I	13 (8.2%)	10 (10.5%)	3 (4.7%)	
II	60 (37.7%)	42 (44.2%)	18 (28.1%)	
III	67 (42.1%)	34 (35.8%)	33 (51.6%)	
Pathological complete response (pcR)				0.2
No pcR	140 (88.1%)	86 (90.5%)	54 (84.4%)	
pcR	19 (11.9%)	9 (9.5%)	10 (15.6%)	
Adjuvant chemotherapy				0.6
Yes	103 (64.8%)	60 (63.2%)	43 (67.2%)	
No	56 (35.2%)	35 (36.8%)	21 (32.8%)	
Adjuvant chemotherapy regimen				0.7
Capecitabine	67 (65.0%)	38 (63.3%)	29 (67.4%)	
Others	36 (35.0%)	22 (36.7%)	14 (32.6%)	
Not reported	56	35	21	
Radiotherapy				0.6
Yes	132 (83.0%)	80 (84.2%)	52 (81.3%)	
No	27 (17.0%)	15 (15.8%)	12 (18.8%)	
Survival				0.5
Alive	104 (65.4%)	60 (63.2%)	44 (68.8%)	
Deceased	55 (34.6%)	35 (36.8%)	20 (31.3%)	
Recurrence				0.1
No recurrence	110 (69.2%)	61 (64.2%)	49 (76.6%)	
Recurrence	49 (30.8%)	34 (35.8%)	15 (23.4%)	

*Note:* The bold values indicate statistically significant results (*p* < 0.05).

^1^Median (Q1, Q3); *n* (%).

^2^Wilcoxon rank sum test; Pearson's Chi-squared test; Fisher's exact test.

**Table 2:** Clinical characteristics associated with pCR.

Clinical characteristics	*N*	OR^1^	95% CI^1^	*p* value
Age (years)	159	1.00	0.96, 1.04	0.8
Age (years)	159			
≤ 40		—	—	
41–59		1.53	0.45, 7.05	0.5
≥ 60		1.48	0.30, 8.04	0.6
Tumor size (cm)	157	0.83	0.67, 1.00	0.071
Clinical stage	159			
II		—	—	
III		0.34	0.11, 0.90	**0.037**
Histological grade	152			
II		—	—	
III		2.81	0.53, 52.1	0.3
HER2 status (diagnosis)	159			
0		—	—	
1+		1.41	0.41, 4.38	0.6
2+		2.39	0.67, 7.73	0.2
HER2 status (diagnosis)	159			
HER2-zero		—	—	
HER2-low		1.77	0.67, 4.73	0.2
Ki67% (diagnosis)	149	1.00	0.98, 1.02	> 0.9
Ki67% (diagnosis)	149			
≤ 20		—	—	
> 20		0.66	0.16, 4.55	0.6

*Note:* The bold values indicate statistically significant results (*p* < 0.05).

^1^OR = odds ratio, CI = confidence interval.

**Table 3:** Impact of HER2 changes on OS and DFS.

Clinical characteristics	OS				DFS			
	*N*	HR^1^	95% CI^1^	*p* value	*N*	HR^1^	95% CI^1^	*p* value
HER2 evolution	117				110			
HER2 0/HER2 0		—	—			—	—	
HER2 0/HER2 low		0.52	0.22, 1.24	0.14		0.48	0.19, 1.20	0.12
HER2 Low/HER2 0		0.9	0.34, 2.40	0.8		0.54	0.18, 1.58	0.3
HER2 Low/HER2 low		0.71	0.34, 1.49	0.4		0.58	0.27, 1.25	0.2

^1^HR = hazard ratio, CI = confidence interval.

## Data Availability

The data used to support the findings of this study are available from the corresponding author upon reasonable request.

## Supporting Information

Table S1: Distribution changes of ER status.

Table S2: Distribution changes of PR status.

Table S3: Distribution changes by HER2 scores.

Table S4: Distribution changes by HER2 status.

Table S5: Clinical characteristics associated with HER2 status conversion. Converted (HER2 0/HER2 LOW or HER2 LOW/HER2 0). Converted (HER2 0/HER2 0 or HER2 LOW/HER2 LOW).

Table S6: Clinical characteristics associated with survival.
