# Supplementary material for: Influence of HER2 Changes on Survival Outcomes After Neoadjuvant Chemotherapy in Peruvian Patients With Triple-Negative Breast Cancer
Source: Breast J. 2025 Nov 14;2025:3770655. doi: 10.1155/tbj/3770655 (PMC12638172; doi:10.1155/tbj/3770655)
Supplement: Supporting Information — Additional supporting information can be found online in the Supporting Information section. [file 3770655.f1.docx]

**Table S1:** Distribution changes of ER status

| **ER status (post-NAC)** | **Overall**  N = 140*^1^* | **ER status (diagnosis)** | | **p-value***^2^* |
| --- | --- | --- | --- | --- |
|  |  | <1% | NEGATIVE |  |
|  |  | N = 8*^1^* | N = 132*^1^* |  |
|  |  |  |  | 0.12 |
| <1% | 5 (3.6%) | 1 (12.5%) | 4 (3.0%) |  |
| 1-10% | 6 (4.3%) | 1 (12.5%) | 5 (3.8%) |  |
| NEGATIVE | 129 (92.1%) | 6 (75.0%) | 123 (93.2%) |  |
| *^1^* n (%) | | | | |
| *^2^* Fisher’s exact test | | | | |

**Table S2:** Distribution changes of PR status

| **PR status (post-NAC)** | **Overall**  N = 140*^1^* | **PR status (diagnosis)** | | **p-value***^2^* |
| --- | --- | --- | --- | --- |
|  |  | <1% | NEGATIVE |  |
|  |  | N = 11*^1^* | N = 129*^1^* |  |
|  |  |  |  | 0.12 |
| <1% | 1 (0.7%) | 0 (0.0%) | 1 (0.8%) |  |
| 1-10% | 5 (3.6%) | 0 (0.0%) | 5 (3.9%) |  |
| NEGATIVE | 134 (95.7%) | 11 (100.0%) | 123 (95.3%) |  |
| *^1^* n (%) | | | | |
| *^2^* Fisher’s exact test | | | | |

**Table S3:** Distribution changes by HER2 scores

| **HER2 score (post-NAC)** | **Overall**, N = 140^1^ | **HER2 score (diagnosis)** | | | **p-value**^2^ |
| --- | --- | --- | --- | --- | --- |
|  |  | **0**, N = 86^1^ | **1+**, N = 34^1^ | **2+**, N = 20^1^ |  |
|  |  |  |  |  | <0.001 |
| 0 | 67 (47.9%) | 54 (62.8%) | 7 (20.6%) | 6 (30.0%) |  |
| 1+ | 54 (38.6%) | 24 (27.9%) | 24 (70.6%) | 6 (30.0%) |  |
| 2+ | 19 (13.6%) | 8 (9.3%) | 3 (8.8%) | 8 (40.0%) |  |
| ^1^n (%) | | | | | |
| ^2^Fisher’s exact test | | | | | |

**Table S4:** Distribution changes by HER2 status

| **HER2 score (post-NAC)** | **Overall**, N = 140^1^ | **HER2 score (diagnosis)** | | **p-value**2 |
| --- | --- | --- | --- | --- |
|  |  | **HER2 0**, N = 86^1^ | **HER2 LOW**, N = 54^1^ |  |
|  |  |  |  | <0.001 |
| HER2-zero | 67 (47.9%) | 54 (62.8%) | 13 (24.1%) |  |
| HER2-Low | 73 (52.1%) | 32 (37.2%) | 41 (75.9%) |  |
| ^1^n (%) | | | | |
| ^2^Pearson's Chi-squared test | | | | |

**Table S5:** Clinical characteristics associated with HER2 status conversion. Converted (HER2 0/HER2 LOW or HER2 LOW/HER2 0). Converted (HER2 0/HER2 0 or HER2 LOW/HER2 LOW)

| **Characteristic** | **Overall** | **Stable** | **Converted** | **p-value***^2^* |
| --- | --- | --- | --- | --- |
|  | N = 140*^1^* | N = 95*^1^* | N = 45*^1^* |  |
| **Age (years)** | 48.0 (41.0, 56.0) | 48.0 (42.0, 57.0) | 49.0 (40.0, 56.0) | 0.8 |
| **Age (years)** |  |  |  | 0.7 |
| ≤ 40 | 31 (22.1%) | 19 (20.0%) | 12 (26.7%) |  |
| 41 - 59 | 81 (57.9%) | 56 (58.9%) | 25 (55.6%) |  |
| ≥ 60 | 28 (20.0%) | 20 (21.1%) | 8 (17.8%) |  |
| **Age (years)** |  |  |  | 0.8 |
| ≤ 40 | 31 (22.1%) | 19 (20.0%) | 12 (26.7%) |  |
| 41 - 59 | 81 (57.9%) | 56 (58.9%) | 25 (55.6%) |  |
| 60 - 69 | 20 (14.3%) | 15 (15.8%) | 5 (11.1%) |  |
| ≥ 70 | 8 (5.7%) | 5 (5.3%) | 3 (6.7%) |  |
| **Histology** |  |  |  | 0.7 |
| Ductal | 129 (92.1%) | 88 (92.6%) | 41 (91.1%) |  |
| Lobular | 3 (2.1%) | 2 (2.1%) | 1 (2.2%) |  |
| Mixed | 2 (1.4%) | 2 (2.1%) | 0 (0.0%) |  |
| Other | 6 (4.3%) | 3 (3.2%) | 3 (6.7%) |  |
| **Tumor size (cm)** | 6.0 (4.0, 8.0) | 6.0 (4.5, 8.0) | 6.0 (4.0, 8.0) | 0.8 |
| Not reported | 2 | 2 | 0 |  |
| **Clinical T** |  |  |  | 0.3 |
| T1 | 3 (2.2%) | 2 (2.2%) | 1 (2.2%) |  |
| T2 | 35 (25.7%) | 21 (23.1%) | 14 (31.1%) |  |
| T3 | 44 (32.4%) | 34 (37.4%) | 10 (22.2%) |  |
| T4 | 54 (39.7%) | 34 (37.4%) | 20 (44.4%) |  |
| Not reported | 4 | 4 | 0 |  |
| **Clinical N** |  |  |  | 0.5 |
| N0 | 30 (27.5%) | 18 (24.7%) | 12 (33.3%) |  |
| N1 | 54 (49.5%) | 39 (53.4%) | 15 (41.7%) |  |
| N2/N3 | 25 (22.9%) | 16 (21.9%) | 9 (25.0%) |  |
| Not reported | 31 | 22 | 9 |  |
| **Clinical stage** |  |  |  | 0.7 |
| II | 59 (42.1%) | 39 (41.1%) | 20 (44.4%) |  |
| III | 81 (57.9%) | 56 (58.9%) | 25 (55.6%) |  |
| **Histological grade** |  |  |  | 0.9 |
| II | 19 (14.2%) | 13 (14.4%) | 6 (13.6%) |  |
| III | 115 (85.8%) | 77 (85.6%) | 38 (86.4%) |  |
| Not reported | 6 | 5 | 1 |  |
| **Ki67% (diagnosis)** | 60.0 (40.0, 70.0) | 60.0 (40.0, 70.0) | 60.0 (40.0, 80.0) | 0.3 |
| Not reported | 9 | 7 | 2 |  |
| **Neoadjuvant chemotherapy regimen** |  |  |  | 0.3 |
| AC-T (no platinum) | 102 (72.9%) | 71 (74.7%) | 31 (68.9%) |  |
| Platinum-containing | 31 (22.1%) | 21 (22.1%) | 10 (22.2%) |  |
| Other | 7 (5.0%) | 3 (3.2%) | 4 (8.9%) |  |
| **Adjuvant chemotherapy regimen** |  |  |  | 0.3 |
| Capecitabine | 67 (66.3%) | 46 (69.7%) | 21 (60.0%) |  |
| Others | 34 (33.7%) | 20 (30.3%) | 14 (40.0%) |  |
| Not reported | 39 | 29 | 10 |  |
| *^1^* Median (Q1, Q3); n (%) | | | | |
| *^2^* Wilcoxon rank sum test; Pearson’s Chi-squared test; Fisher’s exact test | | | | |

**Table S6:** Clinical characteristics associated with survival

| **Clinical characteristics** | **Overall survival (OS)** | | | | **Disease-free survival (DFS)** | | | |
| --- | --- | --- | --- | --- | --- | --- | --- | --- |
|  | **N** | **HR**^1^ | **95% CI**^1^ | **p-value** | **N** | **HR**^1^ | **95% CI**^1^ | **p-value** |
| **Age (years)** | 117 | 1.01 | 0.98, 1.04 | 0.5 | 110 | 1 | 0.97, 1.03 | >0.9 |
| **Age (years)** | 117 |  |  |  | 110 |  |  |  |
| ≤ 40 |  | — | — |  |  | — | — |  |
| 41 - 59 |  | 0.76 | 0.35, 1.63 | 0.5 |  | 1.37 | 0.60, 3.16 | 0.5 |
| ≥ 60 |  | 1.2 | 0.49, 2.96 | 0.7 |  | 0.89 | 0.28, 2.82 | 0.8 |
| **Age (years)** | 117 |  |  |  | 110 |  |  |  |
| ≤ 40 |  | — | — |  |  | — | — |  |
| 41 - 59 |  | 0.76 | 0.35, 1.63 | 0.5 |  | 1.37 | 0.60, 3.15 | 0.5 |
| 60 - 69 |  | 0.81 | 0.27, 2.43 | 0.7 |  | 0.72 | 0.18, 2.77 | 0.6 |
| ≥ 70 |  | 2.31 | 0.77, 6.95 | 0.14 |  | 1.42 | 0.30, 6.87 | 0.7 |
| **Histology** | 117 |  |  |  | 110 |  |  |  |
| Ductal |  | — | — |  |  | — | — |  |
| Lobular/Mixed/Other |  | 0.56 | 0.14, 2.33 | 0.4 |  | 0.76 | 0.18, 3.16 | 0.7 |
| **Tumor size (cm)** | 115 | 1.01 | 0.90, 1.13 | 0.9 | 108 | 1.04 | 0.93, 1.15 | 0.5 |
| **T** | 113 |  |  |  | 106 |  |  |  |
| T1/T2 |  | — | — |  |  | — | — |  |
| T3 |  | 0.54 | 0.24, 1.19 | 0.13 |  | 0.9 | 0.39, 2.09 | 0.8 |
| T4 |  | 1.11 | 0.54, 2.28 | 0.8 |  | 1.18 | 0.51, 2.71 | 0.7 |
| **Clinical stage** | 117 |  |  |  | 110 |  |  |  |
| II |  | — | — |  |  | — | — |  |
| III |  | 0.98 | 0.53, 1.79 | >0.9 |  | 1.04 | 0.56, 1.95 | 0.9 |
| **Histological grade** | 111 |  |  |  | 104 |  |  |  |
| II |  | — | — |  |  | — | — |  |
| III |  | 1.93 | 0.60, 6.27 | 0.3 |  | 0.86 | 0.38, 1.96 | 0.7 |
| **HER2 status (diagnosis)** | 117 |  |  |  | 110 |  |  |  |
| 0 |  | — | — |  |  | — | — |  |
| 1+ |  | 0.69 | 0.31, 1.52 | 0.4 |  | 0.57 | 0.26, 1.26 | 0.2 |
| 2+ |  | 1.5 | 0.68, 3.31 | 0.3 |  | 1.07 | 0.44, 2.62 | 0.9 |
| **Ki67% (diagnosis)** | 109 | 1.01 | 0.99, 1.02 | 0.3 | 102 | 1.01 | 1.00, 1.03 | 0.1 |
| **Surgery type** | 114 |  |  |  | 107 |  |  |  |
| Conservative |  | — | — |  |  | — | — |  |
| Radical |  | 2.3 | 0.97, 5.46 | 0.06 |  | 0.93 | 0.46, 1.87 | 0.8 |
| **HER2 status (post-NAC)** | 117 |  |  |  | 110 |  |  |  |
| 0 |  | — | — |  |  | — | — |  |
| 1+ |  | 0.58 | 0.29, 1.15 | 0.12 |  | 0.56 | 0.27, 1.15 | 0.12 |
| 2+ |  | 0.81 | 0.33, 1.98 | 0.6 |  | 0.78 | 0.29, 2.05 | 0.6 |
| **Ki67% (post-NAC)** | 105 | 1.01 | 1.0, 1.02 | 0.2 | 99 | 1.01 | 1.00, 1.03 | 0.12 |
| **HER2 evolution** | 117 |  |  |  | 110 |  |  |  |
| HER2 0/HER2 0 |  | — | — |  |  | — | — |  |
| HER2 0/HER2 Low |  | 0.52 | 0.22, 1.24 | 0.14 |  | 0.48 | 0.19, 1.20 | 0.12 |
| HER2 Low/HER2 0 |  | 0.9 | 0.34, 2.40 | 0.8 |  | 0.54 | 0.18, 1.58 | 0.3 |
| HER2 Low/HER2 Low |  | 0.71 | 0.34, 1.49 | 0.4 |  | 0.58 | 0.27, 1.25 | 0.2 |
| **Adyuvant chemotherapy** | 117 |  |  |  | 110 |  |  |  |
| Yes |  | — | — |  |  | — | — |  |
| No |  | 1.05 | 0.53, 2.08 | 0.9 |  | 0.71 | 0.32, 1.58 | 0.4 |
| **Radiotherapy** | 117 |  |  |  | 110 |  |  |  |
| Yes |  | — | — |  |  | — | — |  |
| No |  | 1.56 | 0.69, 3.51 | 0.3 |  | 1.22 | 0.51, 2.92 | 0.7 |
| ^1^ HR = Hazard Ratio, CI = Confidence Interval | | | | | | | | |
